# Bilateral Persistent Pupillary Membrane with Tetralogy of Fallot: A Case Report and Review of the Literature

**DOI:** 10.1155/2014/581273

**Published:** 2014-07-24

**Authors:** A. Altun, S. A. Kurna, E. Bozkurt, G. Erdogan, G. Altun, O. O. Olcaysu, S. F. Aki

**Affiliations:** ^1^Clinic of Ophthalmology, Fatih Sultan Mehmet Education and Research Hospital, 34752 Istanbul, Turkey; ^2^Clinic of Ophthalmology, Prof. Dr. N. Resat Belger Beyoglu Eye Education and Research Hospital, 34421 Istanbul, Turkey; ^3^Clinic of Ophthalmology, Umraniye Education and Research Hospital, 34764 Istanbul, Turkey; ^4^Department of Pediatrics, Yeditepe University, 34752 Istanbul, Turkey; ^5^Clinic of Ophthalmology, Erzurum Region Education and Research Hospital, 25240 Erzurum, Turkey

## Abstract

*Case Report*. A 15-year-old boy presented to the Fatih Sultan Mehmet Education and Research Hospital with the complain of bilateral vision blurring associated with severe glare and photophobia. On ophthalmological examination, uncorrected visual acuity was 20/200 in the right eye and 20/100 in the left eye, and there was no improvement with pinhole testing. The slit-lamp examination showed persistent pupillary membranes (PPM) in both eyes. According to the history obtained from his parents, he had received cardiac surgery for tetralogy of Fallot (TOF) 8 years ago. *Conclusion*. This patient is unique because this is the first reported case of bilateral PPM with congenital heart anomaly in the literature. Bilaterality of the eye anomaly strengthens the possibility of an uncommon association between PPM and TOF, rather than local failure in embryonic development.

## 1. Introduction

Persistent pupillary membranes (PPM) are often nonpathogenic physical signs of normal intrauterine development, and they usually regress within the first few weeks of life [1]. PPM may be seen in isolation or in association with other ocular pathologies like microcornea, posterior keratoconus, and macular hypoplasia [2, 3], but they have not been reported with any congenital cardiac anomaly in the literature.

Tetralogy of Fallot (TOF) is the most common form of cyanotic congenital heart disease and is characterized by four distinct anatomic features. Some ocular pathologies like retinal vascular tortuosity, optic disc hypoplasia, trichomegaly, congenital ptosis, strabismus, retinal haemorrhages, prominent eyes, and congenital cataract have been reported with congenital heart disease [4]. In a case report, TOF has been also reported with central retinal artery occlusion [5]. Herein, we report a case of bilateral congenital PPM with TOF. This patient is unique because it is the first reported case with bilateral PPM with congenital heart anomaly in the literature.

## 2. Case Report

A fifteen-year-old boy presented to the Fatih Sultan Mehmet Education and Research Hospital, Istanbul, Turkey, with the complain of vision blurring with severe glare and photophobia in both eyes. He was also suffering from narrow visual field during sunny days and bumping lampposts while walking in the streets.

**Figure 1:**
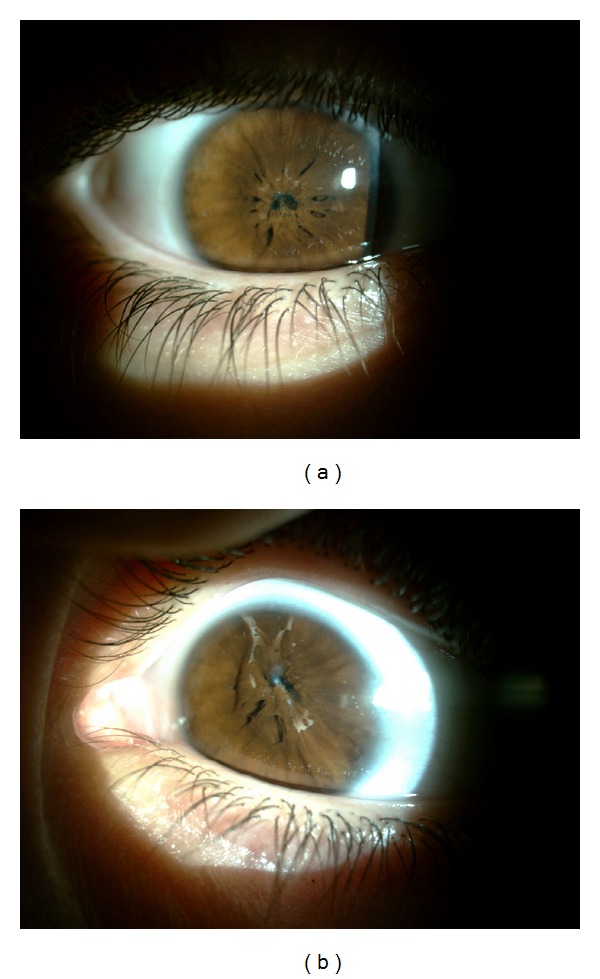
Congenital persistent pupillary membranes were covering the central pupil in the right eye (a) and in the left eye (b).

**Figure 2:**
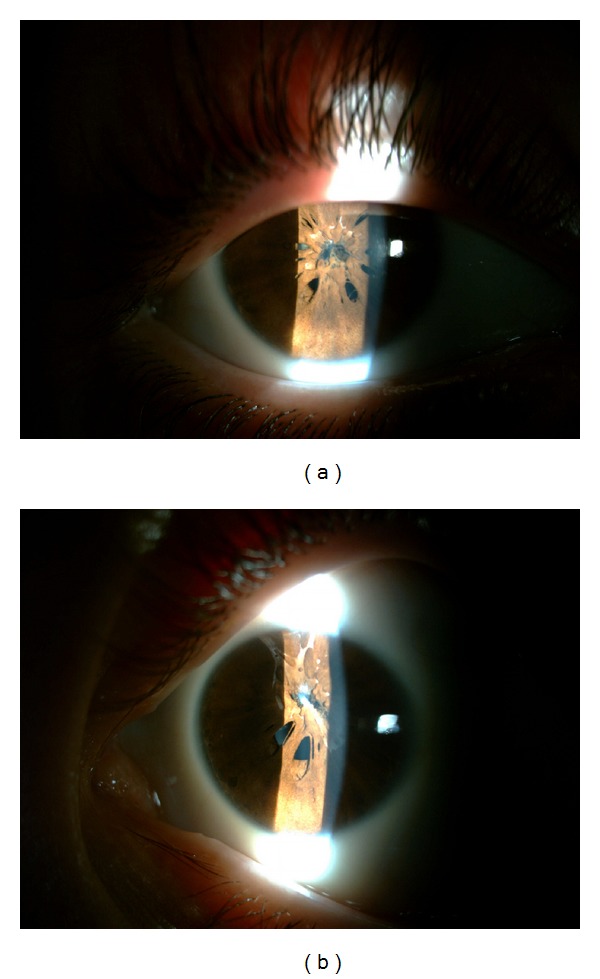
After administration of cyclopentolate eye drops, some parts of lens and fundus were observable and appearing within normal limits in the right eye (a) and in the left eye (b).

On ophthalmological examination, uncorrected visual acuity was 20/200 in the right eye and 20/100 in the left eye, and there was no improvement with pinhole testing. On slit-lamp examination the cornea was clear and the anterior chamber was quite deep in both eyes. Bilateral PPM were covering the central pupil and possibly were adhering to the lens (Figure 1). Clear lens was easily seen partly in the pupillary area not covered with membrane in both eyes. The anterior segment examinations were within normal limits otherwise. After the pupils were dilated by administration of 1% cyclopentolate eye drops, some parts of fundus were observable and appearing within normal limits (Figure 2). Intraocular pressures and gonioscopy findings were within normal limits in both eyes. Horizontal corneal diameter was 10.8 in the right eye and 10.6 mm in the left eye. B-scan ultrasonography revealed normal lens and posterior segment findings in both eyes. Extraocular movements were full and there was no noticed strabismus or nystagmus.

According to the history obtained from his parents, the patient was born of a consanguineous marriage and a normal full-term pregnancy. His mother noticed bilateral PPM when he was a 2-month-old baby. He had no family history of pupil abnormalities or glaucoma and had no other abnormalities such as facial, dental, or umbilical. According to the medical file obtained from the department of pediatrics, he had been following up with the diagnosis of TOF (pulmonary valvular stenosis, ventricular septal defect, overriding aorta, and right ventricular hypertrophy) and had received cardiac surgery when he was 7 years old.

Informed consent was obtained in accordance with the Helsinki Declaration prior to the procedures. The Institutional Review Board approved our review of the patient data. The patient was visually symptomatic and wishing cosmetic and visual correction. The surgical management was performed under general anesthesia in both eyes 2 weeks apart. Two clear corneal 1.5 mm limbal paracenteses were made at the 9 o'clock and 3 o'clock meridians with a microvitreoretinal blade. Sodium hyaluronate 1% was inserted to maintain the anterior chamber and was introduced through the opening in the membrane to raise it apart from the crystalline lens. Vitrectomy probe and microscissors were used to cut PPM. After separation from iris, PPM were gently peeled off from the anterior lens surface with the help of 23-gauge capsulorhexis forceps. Unfortunately, PPM were adhered to the lens surface. During the peeling of the PPMs, anterior lens capsules were damaged and both eyes required crystalline lens extraction. Continuous circular anterior capsulorhexis was performed. After hydrodissection, the crystalline lenses were phacoemulsificated and aspirated through 2.8 mm clear corneal self-sealing corneal incisions. Artificial monocular intraocular lenses were implanted in to the bag without complication.

**Figure 3:**
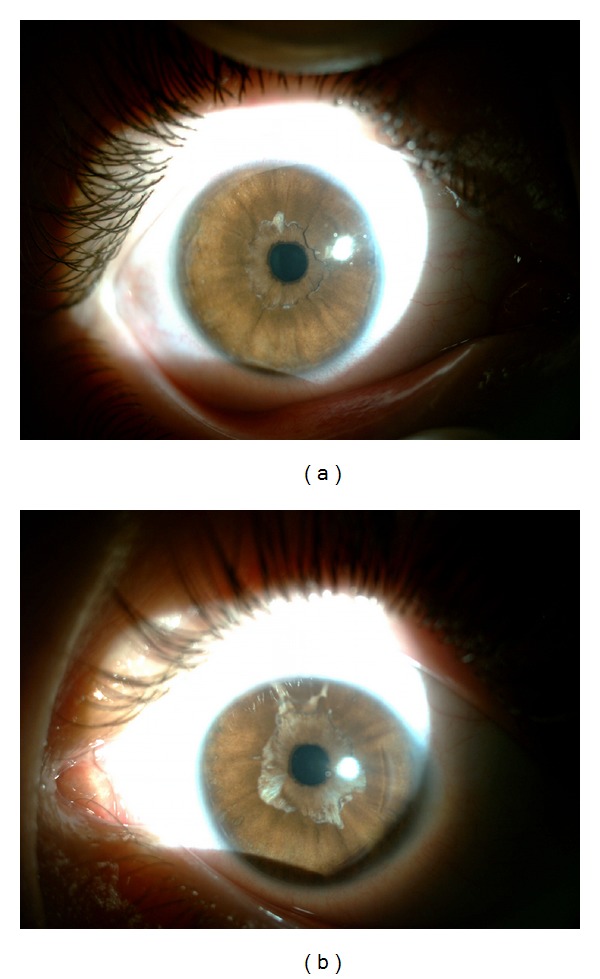
Postoperatively pupillary apertures were adequate and the sphincter muscles were intact and contracting in the right eye (a) and in the left eye (b).

**Figure 4:**
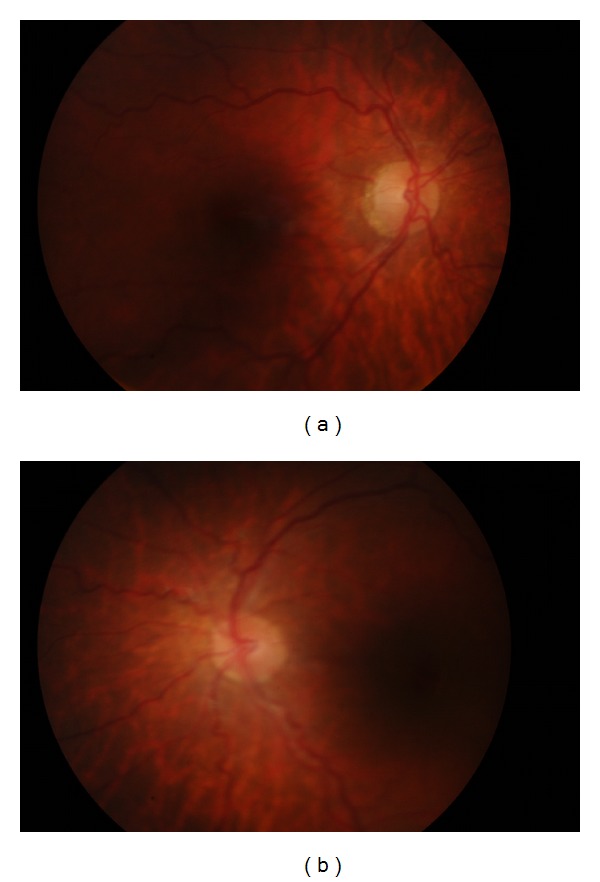
Retinal pigment epithelium alteration and increased venous tortuosity in the right eye (a) and in the left eye (b).

**Figure 5:**
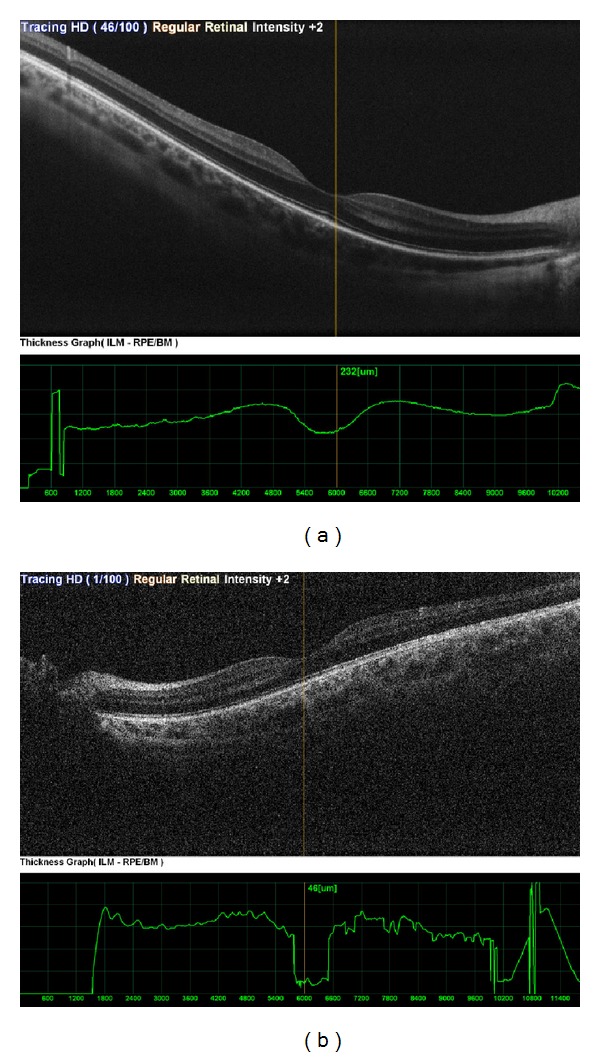
Macular measurements were within normal limits in the right eye (a) and in the left eye (b).

**Figure 6:**
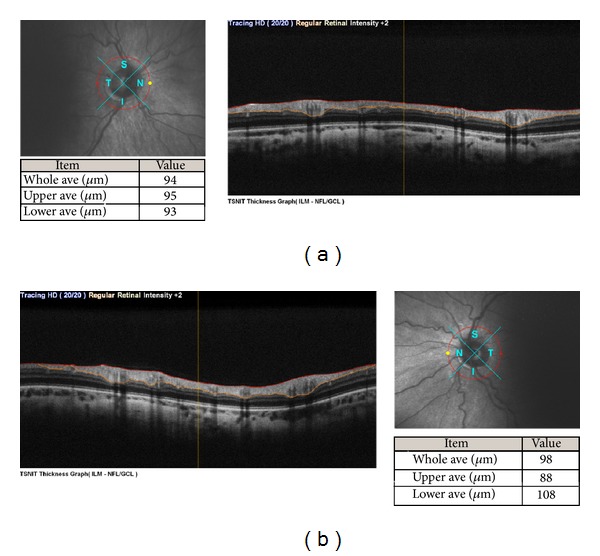
Optic disc measurements were within normal limits in the right eye (a) and in the left eye (b).

His best-corrected visual acuity improved to 20/50 in the right eye and 20/25 in the left eye. Autorefractometer revealed +2.50 − 5.00 × 25 in the right eye and +3.50 + 3.50 × 90 in the left eye. Spectacle correction prescribed was +2.00 − 3.50 × 20 for the right eye and +3.50 + 2.50 × 90 for the left eye. Postoperatively pupillary apertures were adequate and the sphincter muscles were intact and contracting bilaterally (Figure 3). The depth of the anterior chamber and the intraocular pressure were within normal limits bilaterally. Fundus examination revealed retinal pigment epithelium alteration and increased venous tortuosity in both eyes (Figure 4). There was no abnormality in macula (Figure 5) or optic disc (Figure 6) in both eyes when examined with spectral domain optical coherence tomography (Nidek, RS-3000, Japan).

## 3. Discussion

TOF is the most common form of cyanotic congenital heart disease and is characterized by four distinct anatomic features: pulmonary outflow tract obstruction (stenosis or atresia), ventricular septal defect, overriding aortic root, and right ventricular hypertrophy [6]. It is first described in 1888 by the French physician Étienne-Louis Arthur Fallot, after whom it is named. It has an incidence of three per 10,000 live births that accounts for about 5–7% of all congenital heart diseases [7]. The etiology of TOF is multifactorial, but reported associations include untreated maternal diabetes, phenylketonuria, and intake of retinoic acid [8]. Associated chromosomal anomalies can include trisomies [9–11], but recent studies indicate the more frequent association of microdeletions of chromosome 22 [12–14]. Since Blalock performed the first surgery for TOF in 1944 [15], primary repair is now the standard of care and has been safely applied to all age groups [11]. Clinical management is determined by the degree and type of subpulmonary obstruction, in combination with the preference of the centre for the timing of surgical intervention.

PPM are usually nonpathogenic physical sign of normal intrauterine development [1]. The lens is always clear behind the membrane, and the membrane often can be peeled off the lens without causing cataract formation. In our case the membranes were adherent to the lenses; that is why we were unable to remove them without anterior lens capsule damage. Management of the PPM depends on the severity of the presentation. The visual acuity in these patients often remains good despite this remarkable anterior segment anomaly occluding most of the visual axis. This is due to the pinhole effect of apertures within the pupillary membrane. In our patient, the membranes were thick and sticky to the crystalline lenses leading to a significant visual impairment.

The question of which membranes to treat remains a dilemma. If vision is relatively good, some authors recommend conservative management before surgical intervention [2, 16, 17] to avoid the risk of operative complications such as cataract formation. Although our patient had a visual acuity of 20/200 in the right eye and 20/100 in the left eye, the pinhead-sized holes in the membrane were unsatisfactory in size and eccentric in location, giving reason for surgical intervention. The patient was also complaining because of bilateral vision blurring associated with severe glare and photophobia especially in sunny days and wishing for a cosmetic correction. He was also suffering from bumping lampposts while walking in the streets.

All cases need to be evaluated for risk of amblyopia and anisometropia, especially in unilateral cases, for any surgical intervention to ensure the best possible visual acuity. In one study, significant anisometropia was found in over 45 percent of patients [18]. Visually symptomatic cases may be treated with Nd:YAG laser [10, 19, 20] or may require surgical intervention [9, 21–26]. Lambert et al. [27] performed pupilloplasty successfully by using a vitrectomy probe in 4 patients with a clear underlying lens. Some reports of successful surgical removal of symptomatic pupillary membranes without damaging the lens have been reported. Sari et al. [28] reported sodium hyaluronate-assisted pupillary membrane dissection from the lens surface. In our case we had to extract the lenses and implant artificial intraocular lenses bilaterally because of anterior capsule damage due to tight adherence between lenticular membrane and PPM.

For the patients with PPM, good vision can be acquired through reconstructed pupil when amblyopia is not too deep. Our case achieved significant visual improvement after the surgery. His best-corrected visual acuity improved to 20/50 in the right eye and 20/25 in the left eye.

Associations with PPM include amblyopia, cataract, strabismus, and anterior segment abnormalities [29]. Although bilateral familial forms have been reported in the literature [16, 30, 31], most cases are unilateral and sporadic. PPM have not been associated with systemic disorders or any congenital cardiac anomaly. This case is unique because this is the first reported case of bilateral PPM with an emphasis on its possible association with TOF. This case report also emphasizes the importance of ophthalmic examination in the patients with TOF to rule out any vision relevant pathology.
